# Supplementary material for: Predicting Clinical Outcome with Phenotypic Clusters in COVID-19 Pneumonia: An Analysis of 12,066 Hospitalized Patients from the Spanish Registry SEMI-COVID-19
Source: J Clin Med. 2020 Oct 29;9(11):3488. doi: 10.3390/jcm9113488 (PMC7693215; doi:10.3390/jcm9113488)
Supplement: Supplementary file 1 [file jcm-09-03488-s001.pdf]

Supplemental files.

Table S1. Risk factors of ICU admission

	**Multivariate analysis OR (95%CI)***	**p-value**	**Multivariate analysis OR (95%CI)****	**p-value**
**Age/year**	0.97 (0.96-0.98)	<0.001	0.97 (0.96-0.98)	<0.001
**Gender (female)**	0.77 (0.69-0.84)	<0.001	0.74 (0.59-0.92)	0.008
**BMI**	1.02 (1.01-1.03)	<0.001	1.02 (1.01-1.04)	0.040
**Clusters**				
C1	1 ref.		1 ref.	
C2	0.60 (0.51-0.71)	<0.001	0.56 (0.38-0.82)	0.003
C3	1.48 (1.26-1.75)	<0.001	1.48 (1.02-2.14)	0.040
C4	0.94 (0.80-1.10)	0.420	0.93 (0.64-1.34)	0.690
**Comorbidity**				
Arterial hypertension	NS		-	
Diabetes mellitus	1.29 (1.13-1.46)	<0.001	-	
Hyperlipidemia	NS		NS	
COPD	NS		NS	
Ischemic cardiopathy	1.81 (1.52-2.15)	<0.001	1.75 (1.19-2.59)	0.005
Chronic heart failure	0.61 (0.51-0.83)	0.001	NS	
Chronic kidney disease	NS		NS	
Chronic hepatopathy	NS		-	
Active cancer	NS		NS	
**Charlson's index**	0.85 (0.82-0.88)	<0.001	0.85 (0.79-0.91)	<0.001
**Heart rate upon admission**	NS		NS	
**Respiratory rate upon admission >20 bpm**	2.19 (1.99-2.41)	<0.001	2.32 (1.86-2.88)	<0.001
**PaO2/FiO2 upon admission**	0.99 (0.99-0.99)	<0.001	0.99 (0.99-0.99)	<0.001
**Lab test upon admission**				
Lymphocytes $x10^6/l$	NS		NS	
CRP mg/l	NS		NS	
LDH U/l	1.01 (1.01-1.01)	<0.001	1.01 (1.01-1.01)	<0.001
ALT U/l	NS		NS	
Ferritin mcg/l	NS		NS	
D-dimer ng/ml	NS		NS	
**Treatments during admission**				
Remdesivir	5.93 (4.16-8.44)	<0.001	5.96 (4.19-8.47)	<0.001
Tocilizumab	4.33 (3.89-4.82)	<0.001	4.36 (3.92-4.85)	<0.001
Corticosteroids	2.91 (2.64-3.22)	<0.001	2.91 (2.64-3.22)	<0.001

BMI: body mass index. COPD: chronic obstructive pulmonary disease. ALT: alanine transaminase. CRP: C-reactive protein. LDH: lactate dehydrogenase. *All variables included. **Only variables with p<0.10 in the univariate analysis included

Table S2. Risk factors of mechanical ventilation

	Multivariate analysis OR (95%CI)*	p-value	Multivariate analysis OR (95%CI)**	p-value
**Age/year**	0.99 (0.98-0.99)	<0.001	0.99 (0.98-0.99)	0.037
**Gender (female)**	0.80 (0.73-0.87)	<0.001	0.76 (0.63-0.93)	0.009
**BMI**	1.05 (1.04-1.05)	<0.001	1.05 (1.03-1.06)	<0.001
**Clusters**			NS	NS
C1	1 ref.			
C2	0.70 (0.61-0.81)	<0.001		
C3	1.02 (0.87-1.21)	0.773		
C4	1.15 (1.01-1.33)	0.048		
**Comorbidity**				
Arterial hypertension	NS		NS	
Diabetes mellitus	1.17 (1.05-1.31)	0.005	NS	
Hyperlipidemia	NS		NS	
COPD	1.50 (1.30-1.72)	<0.001	1.47 (1.07-2.03)	0.017
Ischemic cardiopathy	1.65 (1.43-1.91)	<0.001	1.66 (1.19-2.31)	0.003
Chronic heart failure	NS		NS	
Chronic kidney disease	NS		NS	
Chronic hepatopathy	0.69 (0.56-0.87)	0.001	-	
Active cancer	NS		-	
**Charlson's index**	0.92 (0.89-0.95)	<0.001	0.92 (0.86-0.97)	0.003
**Heart rate upon admission**	NS		NS	
**Respiratory rate upon admission >20 bpm**	2.20 (2.01-2.39)	<0.001	2.26 (1.86-2.75)	<0.001
**PaO2/FiO2 upon admission**	0.99 (0.99-0.99)	<0.001	0.99 (0.99-0.99)	<0.001
**Lab test upon admission**				
Lymphocytes x10^6/l	NS		NS	
CRP mg/l	NS		NS	
LDH U/l	1.01 (1.01-1.01)	<0.001	1.01 (1.01-1.01)	<0.001
ALT U/l	NS		NS	
Ferritin mcg/l	NS		NS	
D-dimer ng/ml	NS		NS	
**Treatments during admission**				
Remdesivir	7.90 (5.42-11.49)	<0.001	7.52 (3.16-17.90)	<0.001
Tocilizumab	4.59 (4.14-5.07)	<0.001	4.68 (3.71-5.91)	<0.001
Corticosteroids	3.29 (3.01-3.60)	<0.001	3.30 (2.69-4.04)	<0.001

BMI: body mass index. COPD: chronic obstructive pulmonary disease. ALT: alanine transaminase. CRP: C-reactive protein. LDH: lactate dehydrogenase. *All variables included. **Only variables with p<0.10 in the univariate analysis included
